# Association of bovine leptin polymorphisms with energy output and energy storage traits in progeny tested Holstein-Friesian dairy cattle sires

**DOI:** 10.1186/1471-2156-11-73

**Published:** 2010-07-29

**Authors:** Linda Giblin, Stephen T Butler, Breda M Kearney, Sinead M Waters, Michael J Callanan, Donagh P Berry

**Affiliations:** 1 Teagasc, Food Research Centre, Moorepark, Fermoy, Co. Cork, Ireland; 2 Animal and Bioscience Research Department, Animal and Grassland Research and Innovation Centre, Teagasc, Moorepark, Fermoy, Co.Cork, Ireland; 3 Biochemistry Department, University College Cork, Cork, Ireland; 4 Animal and Bioscience Research Department, Animal and Grassland Research and Innovation Centre, Teagasc, Grange, Dunsany, Co. Meath, Ireland

## Abstract

**Background:**

Leptin modulates appetite, energy expenditure and the reproductive axis by signalling via its receptor the status of body energy stores to the brain. The present study aimed to quantify the associations between 10 novel and known single nucleotide polymorphisms in genes coding for leptin and leptin receptor with performance traits in 848 Holstein-Friesian sires, estimated from performance of up to 43,117 daughter-parity records per sire.

**Results:**

All single nucleotide polymorphisms were segregating in this sample population and none deviated (P > 0.05) from Hardy-Weinberg equilibrium. Complete linkage disequilibrium existed between the novel polymorphism LEP-1609, and the previously identified polymorphisms LEP-1457 and LEP-580. LEP-2470 associated (P < 0.05) with milk protein concentration and calf perinatal mortality. It had a tendency to associate with milk yield (P < 0.1). The G allele of LEP-1238 was associated (P < 0.05) with reduced milk fat concentration, reduced milk protein concentration, longer gestation length and tended to associate (P < 0.1) with an increase in calving difficulty, calf perinatal mortality and somatic cells in the milk. LEP-963 exhibited an association (P < 0.05) with milk fat concentration, milk protein concentration, calving difficulty and gestation length. It also tended to associate with milk yield (P < 0.1). The R25C SNP associated (P < 0.05) with milk fat concentration, milk protein concentration, calving difficulty and length of gestation. The T allele of the Y7F SNP significantly associated with reduced angularity (P < 0.01) and reduced milk protein yield (P < 0.05). There was also a tendency (P < 0.1) for Y7F to associate with increased body condition score, reduced milk yield and shorter gestation (P < 0.1). A80V associated with reduced survival in the herd (P < 0.05).

**Conclusions:**

Several leptin polymorphisms (LEP-2470, LEP-1238, LEP-963, Y7F and R25C) associated with the energetically expensive process of lactogenesis. Only SNP Y7F associated with energy storage. Associations were also observed between leptin polymorphisms and calving difficulty, gestation length and calf perinatal mortality. The lack of an association between the leptin variants investigated with calving interval in this large data set would question the potential importance of these leptin variants, or indeed leptin, in selection for improved fertility in the Holstein-Friesian dairy cow.

## Background

Leptin is a 16 kD non-glycosylated polypeptide that signals the body fat reserves and energy status to the hypothalamus. It is primarily produced by adipose tissue and acts to alter feed intake [1,2]. Leptin binds to its receptor mainly localised on neuropeptide Y-neurons, which results in an increase in energy output and hypophagia [3,4]. Leptin acts as a body barometer providing a critical link between energy homeostasis, appetite and reproductive function [4-6]. During early lactation, dairy cows are in a state of negative energy balance where fat stores and feed intake are directed towards the energetically expensive process of lactogenesis [7]. Reproduction, therefore, receives a low priority [5,7-9]. With a noticeable decline in fertility in the modern dairy cow as a result of the intense selection for milk production alone [10], knowledge of combination allelic variations in the bovine leptin gene, or its receptor, may provide a mechanism to select for improved fertility without negatively affecting appetite or milk production.

The bovine leptin gene (*LEP*) maps to chromosome 4q32, is 16.735 kb in length, spans 3 exons and codes for a protein of 167 amino acids which includes a 21 amino acid signal sequence [11,12]. The bovine *LEP *promoter stretches approximately 3 kb upstream [12]. To date, polymorphisms in the bovine *LEP *gene locus have been associated with serum leptin concentrations [13,14], dry matter intake [15], feed intake [13,15-17], milk energy output [15,18-20] and energy storage [21-24]. The pleiotropic effects of leptin are further evidenced by associations between *LEP *polymorphisms and immune function [19,25], calf prenatal mortality [26], calving interval [27], carcass traits [13], lean meat yield [23] and growth rate [13,28].

The bovine leptin receptor gene (*LEPR*) is located on chromosome 3q33 [29]. A polymorphism in exon 20 (T945M) associates with serum leptin levels in late pregnancy in Holstein-Friesian dairy cows [30]. Although no associations have been found with milk yield and feed intake in other cattle breeds [15], associations have been found with *LEPR *polymorphisms and subcutaneous fat deposition, carcass fat yield [31] and growth [32]. In addition, polymorphisms in *LEPR *in other species have been linked to reproductive performance [33] and body mass [34].

The present study aimed to quantify the associations between novel and previously reported single nucleotide polymorphisms (SNP) in the *LEP *and *LEPR *with performance traits in Holstein-Friesian sires.

## Results and Discussion

### SNP discovery and detection in the Holstein-Friesian study population

Sequence analysis of 1.656 kb of the regulatory region of *LEP *in 14 Holstein-Friesian sires, selected based on their predicted transmitting ability for body condition score (BCS) being greater than 2 standard deviation units each side of the base population, revealed a T to C substitution at position -1609 bp upstream of the transcription start site (LEP-1609).

A total of 848 Holstein-Friesian sires were genotyped for this novel and 9 previously identified SNPs (LEPR-T945M, LEP-2470, LEP-1457, LEP-1238, LEP-963, LEP-580, Y7F, R25C and A80V). The performance traits evaluated are not expressed by the sires themselves, but rather by their female progeny. Statistical analysis of the female progeny facilitates the estimation of genetic merit on individual sires. Systematic environmental effects are adjusted for and the random non-genetic variation associated with individual animal phenotypes is minimised, thereby resulting in a more accurate phenotype compared to the phenotype on the individual itself. This increased study power is particularly beneficial for low heritability traits such as fertility where the proportion of phenotypic variance attributable to additive genetic differences is low. The disadvantage of such a study design is that the performance traits included in this analysis are limited to traits routinely measured on progeny. In the present study the average number of progeny per sire was 842 daughter-parity records. The average co-ancestry among the 848 sires was 2.2%. When coupled with the mixed model methodology used and the de-regression of the predicted transmitting abilities (PTA), this implies that the associations reported herein are independent of pedigree structure.

Table 1 details the linkage disequilibrium (r^2^) between the *LEP *SNPs. Due to the complete linkage disequilibrium between phases among the SNPs LEP-1609, LEP-1457 and LEP-580, only LEP-1457 is reported in the association analysis. In addition, the strong linkage disequilibrium between SNPs LEP-1238, LEP-963 and R25C suggests that these SNPs are unlikely to act independently of each other. Indeed Banos *et al. *(2008) reported strong linkage disequilibrium between LEP-963 and R25C [15]. Table 2 lists the genotype and allele frequencies of *LEPR *and the remaining 7 *LEP *polymorphisms. All SNPs were segregating in this sample population and none deviated (P > 0.05) from Hardy-Weinberg equilibrium. The minor allele frequency of the SNPs varied from 0.04 to 0.48 (Table 2). Homozygous TT at position Y7F was not present in the dataset.

**Table 1:** Linkage disequilibrium (r^2^) between the SNPs in the promoter and coding region of the leptin gene

SNP	LEP-2470	LEP-1609	LEP-1457	LEP-1238	LEP-963	LEP-580	Y7F	R25C
**LEP-1609**	0.14							
**LEP-1457**	0.14	1.00						
**LEP-1238**	0.09	0.62	0.62					
**LEP-963**	0.09	0.62	0.62	0.96				
**LEP-580**	0.14	1.00	1.00	0.62	0.62			
**Y7F**	0.01	0.02	0.02	0.04	0.04	0.02		
**R25C**	0.08	0.61	0.61	0.95	0.96	0.61	0.07	
**A80V**	0.06	0.38	0.38	0.23	0.23	0.38	0.01	0.24

**Table 2:** Genotype frequencies, minor allele frequencies (MAF) and significance of deviation from Hardy-Weinberg equilibrium (HWE) for the leptin and leptin receptor SNPs

Region	GenBank Accession No.	SNP	BTA	Position	Genotype	Genotype Frequency (%)	MAF (%)	**HWE**^**1**^
Receptor	AJ580801	LEPR-T945M	3	85691334	C/C	0.83	0.09	0.66
					C/T	0.17		
					T/T	0.01		
Promoter	AB070368	LEP-2470	4	95675400	C/C	0.75	0.13	0.25
					C/T	0.24		
					T/T	0.01		
Promoter		LEP-1457	4	95676411	A/A	0.27	0.48	1.00
					A/G	0.50		
					G/G	0.23		
Promoter		LEP-1238	4	95676628	C/C	0.14	0.37	0.57
					C/G	0.45		
					G/G	0.40		
Promoter		LEP-963	4	95676903	C/C	0.41	0.37	0.75
					C/T	0.46		
					T/T	0.14		
Coding	U50365	Y7F	4	95689996	A/A	0.93	0.04	0.08
		(dsSNP rs29004487)			A/T	0.07		
					T/T	0.00		
Coding		R25C	4	95690050	C/C	0.40	0.37	0.78
		(dsSNP rs29004488)			C/T	0.47		
					T/T	0.13		
Coding		A80V	4	95691973	C/C	0.49	0.29	0.05
		(dsSNP rs29004508)			C/T	0.44		
					T/T	0.07		

^1^Significance of the deviation from HWE derived using a chi-square test

LEP-580 and LEP-1609 were omitted from the table as they exhibit complete linkage disequilbrium with LEP-1457

### Association analysis

Allele substitution effects of the 8 SNPs (LEPR-T945M, LEP-2470, LEP-1457, LEP-1238, LEP-963, Y7F, R25C and A80V) on the energy intensive processes of reproduction and lactogenesis are detailed in Tables 3 and 4. Table 5 describes the associations between the 8 SNPs and energy storage. *LEP *polymorphisms exhibited associations with milk composition, angularity, calving difficulty, gestation length, calf perinatal mortality and survival in the herd. LEPR-T945M did not associate with any of the traits examined.

**Table 3:** Allele substitution effect (standard error in parenthesis) between the 8 SNPs and survival in the herd, reproduction and calving performance

SNP	Allele	Survival in the herd (%)	Calving interval (days)	Direct calving difficulty (%)	Maternal calving difficulty (%)	Gestation length (days)	Calf Perinatal mortality (%)
**Number of bulls**		477	501	575	506	614	201
**LEPR-T945M**	C to T	-0.2833 (0.2395)	0.20 (0.44)	0.26 (0.20)	0.08 (0.25)	-0.06 (0.13)	-0.06 (0.16)
**LEP-2470**	C to T	-0.1036 (0.2060)	0.19 (0.37)	-0.02 (0.17)	0.11 (0.21)	-0.05 (0.11)	0.30 (0.13)*
**LEP-1457**	A to G	0.2072 (0.1290)	-0.07 (0.23)	-0.11 (0.12)	-0.07 (0.13)	-0.11 (0.07)	-0.12 (0.08)
**LEP-1238**	C to G	-0.2009 (0.1361)	0.07 (0.25)	0.24 (0.12)†	-0.12 (0.14)	0.16 (0.08)*	0.16 (0.09) †
**LEP-963**	C to T	0.1737 (0.1333)	-0.05 (0.24)	-0.30 (0.12)*	0.2 (0.14)	-0.17 (0.08)*	-0.14 (0.09)
**Y7F**	A to T	-0.1500 (0.3342)	0.11 (0.60)	-0.22 (0.28)	0.25 (0.33)	-0.33 (0.18) †	0.10 (0.23)
**R25C**	C to T	0.1550 (0.1348)	0.03 (0.24)	-0.28 (0.12)*	0.16 (0.14)	-0.18 (0.08)*	-0.10 (0.09)
**A80V**	C to T	-0.3049 (0.1476)*	0.29 (0.27)	0.11 (0.13)	0.00 (0.15)	0.05 (0.08)	0.08 (0.10)

Significance of difference from zero^† ^= P < 0.10; * = P < 0.05; ** = P < 0.01

Because of complete linkage disequilibrium between phases among the SNPs LEP-1457, LEP-1609 and LEP-580, only LEP-1457 was retained for the association analysis

**Table 4:** Allele substitution effect (standard error in parenthesis) between the 8 SNPs and milk production

SNP	Allele	Milk yield (kg)	Fat yield (kg)	Protein yield (kg)	Fat percent (%)	Protein percent (%)	**SCS (log**_**e **_**units)**
**Number of bulls**		742	742	742	742	742	703
**LEPR-T945M**	C to T	-21.49 (16.8)	-0.53 (0.60)	-0.60 (0.47)	0.0067 (0.0127)	0.0033 (0.0062)	0.0009 (0.0114)
**LEP-2470**	C to T	23.61 (14.29)^†^	0.05 (0.51)	0.20 (0.40)	-0.0172 (0.0108)	-0.0114 (0.0052)*	0.0006 (0.0096)
**LEP-1457**	A to G	-7.73 (9.16)	0.16 (0.32)	0.00 (0.25)	0.0072 (0.0069)	0.0045 (0.0033)	-0.0097 (0.0061)
**LEP-1238**	C to G	14.57 (9.87)	-0.38 (0.35)	0.03 (0.27)	-0.0159 (0.0073)*	-0.0080 (0.0035)*	0.0106 (0.0065)^†^
**LEP-963**	C to T	-16.29 (9.62)^†^	0.27 (0.34)	-0.15 (0.27)	0.0154 (0.0072)*	0.0073 (0.0035)*	-0.0079 (0.0064)
**Y7F**	A to T	-39.19 (22.70)^†^	-1.11 (0.80)	-1.25 (0.63)*	0.0056 (0.0170)	-0.0007 (0.0083)	-0.0152 (0.0153)
**R25C**	C to T	-13.32 (9.61)	0.36 (0.34)	-0.07 (0.27)	0.0152 (0.0072)*	0.0071 (0.0035)*	-0.0100 (0.0064)
**A80V**	C to T	7.97 (10.55)	-0.12 (0.38)	0.21 (0.29)	-0.0073 (0.0079)	-0.0009 (0.0039)	0.0105 (0.0070)

Significance of difference from zero ^† ^= P < 0.10; * = P < 0.05

Because of complete linkage disequilibrium between phases among the SNPs LEP-1457, LEP-1609 and LEP-580, only LEP-1457 was retained for the association analysis

**Table 5:** Allele substitution effect (standard error in parenthesis) between the 8 SNPs and body fatness

SNP	Allele	Angularity (SD Units)	BCS (SD Units)	Carcass fat (Scale 1-15)
**Number of bulls**		521	504	446
**LEPR-T945M**	C to T	0.12 (0.15)	0.11 (0.12)	-0.0338 (0.0303)
**LEP-2470**	C to T	0.14 (0.13)	-0.06 (0.10)	0.0052 (0.0261)
**LEP-1457**	A to G	0.05 (0.08)	-0.01 (0.07)	0.0058 (0.0164)
**LEP-1238**	C to G	-0.03 (0.09)	0.04 (0.07)	-0.0051 (0.0180)
**LEP-963**	C to T	0.08 (0.09)	-0.06 (0.07)	0.0009 (0.0174)
**Y7F**	A to T	-0.74 (0.23)**	0.32 (0.19) ^†^	0.0276 (0.0392)
**R25C**	C to T	0.06 (0.09)	-0.05 (0.07)	-0.0042 (0.0178)
**A80V**	C to T	0.05 (0.10)	-0.06 (0.08)	-0.0094 (0.0186)

Significance of difference from zero ^† ^= P < 0.10; * = P < 0.05; ** = P < 0.01

Because of complete linkage disequilibrium between phases among the SNPs LEP-1457, LEP-1609 and LEP-580, only LEP-1457 was retained for the association analysis

BCS: Body Condition Score, SD Units: Standard Deviation units

### Reproduction

Leptin interacts with the reproductive axis at multiple sites [35] and is a key regulator in foetal development [36]. The G allele of LEP-1238 was associated (P < 0.05) with an increase in gestation length and tended (P < 0.10) to be associated with an increase in calving difficulty and calf perinatal mortality (Table 3). Previously, Brickell *et al*. reported an association in heifers between calf perinatal mortality and SNP UASMS1, a SNP in complete linkage disequilibrium with LEP-1238 [13,26]. Leifers *et al*. observed that the G allele at LEP-1238 tended to associate with a quicker return to luteal activity postpartum [20]. However, in our data set calving interval is unaffected (Table 3). LEP-963 T allele was associated (P < 0.05) with easier calving and shorter gestation length (Table 3). Interestingly, Leifers *et al*. noted a longer interval from calving to 1^st ^observed oestrus in cows with the T allele at LEP-963 [20].

The T allele of R25C, which associated with shorter gestation length and easier calving in our study (Table 3), was associated with lower serum leptin protein concentrations during late pregnancy and higher calf perinatal mortality in Holstein-Friesians [14,26]. In humans, leptin is thought to play an inhibitory role in uterine contractions and has been suggested as a tocolytic agent for threatened preterm [37]. It is possible that where maternal leptin concentration is low, a preterm labour (short gestation) may occur resulting in an easier birthing event of a smaller birth weight calf with an increased risk of perinatal mortality.

The associations with gestation length remained significant or approached significance when each of the three SNPs (LEP-1238, LEP-963 and R25C) was included in the model as a class variable (additional file 1). Using this alternative analysis, calving difficulty also significantly associated with LEP-963 and tended to associate with R25C. To the authors knowledge this is the first analysis of *LEP *polymorphisms with either gestation length or calving difficulty. What role leptin plays in gestation length or a parturition event remains to be determined.

The T allele of LEP-2470 was associated (P < 0.05) with an increase in calf perinatal mortality (Table 3). When the genotype was included in the model as a class effect, this association approached significance (P < 0.1, see additional file 1). However, Brickell *et al. *[26] reported no association between this SNP and calf perinatal mortality in 381 Holstein-Friesian nulliparous heifers. Our study uses calf perinatal data from multiparious cows in addition to heifers, which may explain the discrepancy. Furthermore, despite the fewer number of records used in our study, the accuracy of the phenotype for calf mortality is likely to be greater since PTA was used here which is based on performance in daughter descendants.

Interestingly, there was no corresponding association observed between LEP-2470 and calving difficulty (maternal and direct) which suggests that fetopelvic incompatibility does not contribute to the mortality association observed with LEP-2470. Although LEP-2470 may not be the causative mutation, this SNP lies in a sequence conserved in human, rat and bovine leptin promoters and adjacent to a putative binding site for estrogen binding protein (E2BP) transcription factor. Allele specific differences in the E2BP binding matrix may influence estrogen regulation of *LEP *expression [38]. Previously, associations have been found with the T allele and increased serum leptin concentrations, backfat and growth rate in beef cattle [13,28]. It is possible that the T allele is also linked to similar phenotypes in the dairy cow which may partition nutrients away from the developing fetus with a detrimental effect on calf perinatal survival [26].

None of the *LEP *polymorphisms selected in the present study associated with fertility as measured by calving interval i.e. the number of days between consecutive parturitions. In particular, no association was observed between SNP-1457 and calving interval (Table 3), even though AG heterozygotes of LEP-1457 commenced luteal activity 7.1 days earlier postpartum than AA homozygotes [20]. However, calving interval has a lower heritability than commencement of luteal activity postpartum [39] and is a more complex phenotype influenced not only by luteal activity but also fertilization, embryo implantation and embryo survival. In the present study calving interval and gestation length are not confounded. Gestation length in the present study is based on the direct effects of the sire on the calf itself while calving interval is based on the direct effects of the sire through its expression in his daughters (i.e., the maternal grandsire of the calf). There is a part-whole relationship between calving interval and maternal gestation length and as such PTAs for the latter are not calculated in Ireland.

In the present study, A80V associated with survival in the herd (Table 3). Survival is influenced by several factors including fertility, health, milk production and temperament. Previous work in other data sets have demonstrated associations between the T allele and increased serum leptin concentrations in late pregnancy [14], increased milk yield and protein yield [18], more rapid growth rates and heavier animals [22]. Although this SNP has no association with milk, carcass or fertility traits individually in our data set, there may be an additive effect resulting in the observed association with survival.

### Milk Production

Although circulating leptin concentrations are low in early lactation [14], leptin is locally produced by the mammary gland [40] and appears to play important roles in mammary gland development [41] and lactogenesis [42,43]. The T allele of LEP-2470 associated (P < 0.05) with reduced milk protein concentration and had a tendency to associate with increased (P < 0.10) milk yield (Table 4). In agreement with Banos *et al. *[15], the tendency of this SNP to associate with milk yield in Holstein-Friesian cattle does not persist in our study when the genotype is included in the model as a class effect. To the authors' knowledge, this is the first time this SNP has been tested and found to associate with milk protein.

SNPs LEP-1238, LEP-963 and R25C significantly associated with milk fat and milk protein concentration (Table 4). In addition, the G allele of LEP-1238 tended (P < 0.10) to associate with an increase in somatic cell score (SCS) in the milk and the T allele of LEP-963 tended to associate with reduced milk yield (P < 0.10). When the genotypes were included as class effects in the model, the association with milk fat concentration for each of the three SNPs approached significance (P < 0.10). However the association with protein concentration only approached significance for LEP-1238 (additional file 1). Although none of the three SNPs may be the causative mutation for the observed association to milk composition, potential mechanisms for this association may lie in the location of LEP-1238 in a putative binding site for CCAAAT/enhancer binding protein (C/EBP). The polymorphism may alter the binding affinity of C/EBP at this locus. C/EBP sites along the promoter, in other species, play an important role in regulating *LEP *transcription [44,45]. In a dose dependant manner, leptin upregulates the lactogenic effect of prolactin, enhancing induction of fatty acid synthesis and milk protein gene expression [43].

Previous association analyses with LEP-1238 and LEP-963 did not find an association with milk composition or yield [15,20]. In addition some conflicting evidence exists for the association of R25C with milk production. The T allele at R25C associated with increased milk yield, milk protein yield and decreased milk fat concentration [46]. CT heterozygous Holstein cows had greater yields of milk fat and milk protein compared to CC cows [19]. In other data sets no associations were found between R25C and milk production traits in either Polish Black and White or Holstein cattle [15,18]. The discrepancy between our data and these studies could be due to a multitude of factors: 1) the frequency of the different alleles in the sample populations; 2) the experimental design (e.g., using estimated breeding value or actual phenotypes as the dependent variable) or the statistical models and data used (e.g., parity of the animal or period during which the phenotype was measured), 3) genotype by environment interaction or 4) the genetic background of the animals.

The rare T allele of SNP T7F associated with a decrease in protein yield (P < 0.05) and tended to associate with a decrease in milk yield (P < 0.10) (Table 4). However these associations did not hold when the genotype was analyzed as a class effect (additional file 1).

### Energy Storage

Angularity in the dairy cow is a subjective measure of adiposity [47]. Only SNP T7F exhibited a significant association with angularity (P < 0.01; Table 5). It also tended to associate with BCS (P < 0.10), a trait genetically similar yet opposite to angularity (Berry et al., 2004). Indeed when the genotype was analyzed as a class effect, the significant association (P < 0.05) with angularity remained (additional file 1). This agrees with previous work which indicated an association between this SNP and BCS [15,16,23]. T7F results in a tyrosine to phenylalanine substitution at amino acid position 7 within the peptide signal sequence. This substitution may alter processing and export of leptin, disrupting its ability to report on levels of adiposity.

LEP-1238, LEP-963 and R25C did not associate with carcass fat, angularity or BCS (Table 5) in our study. Previously, LEP-1238 exhibited associations with energy balance, feed intake and body weight but there is some conflicting evidence about LEP-963 and these traits in various data sets [13,15,20]. The T allele of R25C was associated with significantly heavier calves at weaning, fatter carcasses, slower growth rates, and higher leptin mRNA levels in adipose tissue compared to the C allele, albeit in beef cattle [28,48,23,52].

### Haplotype analysis

Using the SNPs in the promoter and coding region of *LEP*, 22 possible haplotypes were reconstructed; 16 haplotypes occurred in less than 1% in the population and were therefore grouped together for the analysis (Table 6). None of the haplotypes were associated with fertility. Haplotype C_-2470_G_-1457_G_-1238_C_-963_A_Y7F_C_R25C_C_A80V _was associated with an increase in milk fat yield of 1.22 kg, milk protein yield of 0.97 kg and milk protein concentration of 0.0128 percentage units. No adverse associations with milk yield, calving interval, functional survival, carcass fat or calf perinatal mortality were observed. Interestingly, this haplotype contains a G allele at LEP-1238, a C allele at LEP-963 and a C allele at R25C, all of which associated with a decrease in protein concentration. The C allele at LEP-2470, which associated with an increase in protein concentration, therefore appears to overpower the influence of these other alleles on milk protein in this haplotype. The two major haplotypes C_-2470_G_-1457_C_-1238_T_-963_A_Y7F_T_R25C_C_A80V _and C_-2470_A_-1457_G_-1238_C_-963_A_Y7F_C_R25C_T_A80V _represent 62% of the population and only differ in genetic merit for milk protein concentration. Three minor haplotypes (12%, 11% and 3%) were associated with an increase in angularity compared to their major counterparts.

**Table 6:** Regression coefficients (standard errors in parenthesis) of the performance traits on the different haplotypes

Trait				Haplotype			
	CGCTATC	CAGCACT	TAGCACC	CGGCACC	CAGCACC	CGCTTTC	Other
**Frequency **(%)	33	29	12	11	10	3	2
**Milk yield **(kg)	0^ab^	-39.97 (24.06)^a^	30.35 (26.77)^ab^	8.56 (25.83)^ab^	-4.92 (12.66)^ab^	59.46 (34.3)^b^	-65.36 (52.68)^a^
**Fat yield **(kg)	0^ab^	-1.07 (0.86)^ab^	0.12 (0.95)^ab^	1.22 (0.92)^a^	0.03 (0.45)^ab^	0.37 (1.22)^ab^	-3.23 (1.88)^b^
**Protein yield **(kg)	0^ab^	-0.55 (0.67)^ab^	0.87 (0.75)^a^	0.97 (0.72)^a^	0.21 (0.35)^ab^	1.01 (0.95)^a^	-2.48 (1.46)^b^
**Fat percent **(%)	0	0.0131 (0.0182)	-0.0223 (0.0203)	0.0117 (0.0195)	0.0020 (0.0096)	-0.0417 (0.0259)	-0.0231 (0.0396)
**Protein percent **(%)	0^ac^	0.0188 (0.0088)^b^	-0.0021 (0.0098)^abc^	0.0128 (0.0094)^bc^	0.0064 (0.0046)^abc^	-0.0193 (0.0125)^a^	-0.0093 (0.0192)^abc^
**SCS **(units)	0	0.0191 (0.0147)	0.0265 (0.0162)	0.0084 (0.0157)	-0.0009 (0.0077)	0.0063 (0.0208)	-0.0166 (0.0327)
**Calving interval **(days)	0	-0.50 (0.61)	0.79 (0.68)	0.52 (0.65)	-0.49 (0.33)	0.74 (0.87)	0.24 (1.37)
**Survival **(%)	0	0.1442 (0.3382)	-0.4526 (0.3793)	0.1187 (0.3632)	-0.0364 (0.1817)	-0.2627 (0.4888)	-0.2832 (0.7717)
**Perinatal mortality **(%)	0	0.25 (0.22)	0.02 (0.27)	-0.16 (0.25)	-0.13 (0.13)	0.48 (0.31)	-0.20 (0.53)
**Calving difficulty **(%)	0	-0.107(0.2947)	-0.1667(0.344)	-0.6468(0.3347)	-0.0046(0.1608)	-0.2876(0.4124)	-0.6397(0.6402)
**Gestation length **(days)	0^a^	0.1288(0.1826)^a^	-0.2196(0.2086)^ac^	-0.4698(0.2086)^bc^	0.0096(0.0978)^a^	-0.371(0.2597)^ab^	-0.996(0.3961)^bc^
**Maternal calving difficulty **(%)	0	0.0856(0.3495)	0.3612(0.3856)	0.6681(0.3808)	0.1923(0.1856)	0.7648(0.488)	0.6698(0.7485)
**BCS **(SD units)	0^ab^	0.09 (0.20)^a^	-0.33 (0.21)^a^	-0.35 (0.19)^b^	0.08 (0.09)^a^	-0.34 (0.25)^a^	0.53 (0.45)^a^
**Angularity **(SD units)	0^a^	-0.37 (0.25)^a^	0.56 (0.26)^b^	0.70 (0.24)^b^	-0.07 (0.12)^a^	0.79 (0.31)^b^	-0.95 (0.53)^a^
**Carcass fat **(Scale 1-15)	0^a^	0.0415 (0.0432)^ab^	-0.0285 (0.0482)^ab^	-0.0104 (0.0478)^ab^	0.0462 (0.0235)^b^	0.0044 (0.0622)^ab^	0.0428 (0.0874)^ab^

Polymorphism order: LEP-2470, LEP-1457, LEP-1238, LEP-963, Y7F, R25C and A80V

^† ^CGCTATC haplotype taken to represent the referent haplotype

^a,b,c ^Regression coefficients with different superscript letters are different (P < 0.05) from each other

BCS: Body Condition Score, SDU: standard deviation units

## Conclusions

Of the 10 *LEP *and *LEPR *SNPs tested, LEP-2470, LEP-1238, LEP-963, Y7F and R25C associated with the energetically expensive process of lactogenesis. Animals with the C_-2470_G_-1457_G_-1238_C_-963_A_Y7F_C_R25C_C_A80V _*LEP *haplotype had a superior genetic merit for milk fat yield, protein yield and milk protein concentration. SNP Y7F associated with energy storage. Several *LEP *polymorphisms associated with calving difficulty, gestation length or calf perinatal mortality. However, the absence of an association between the *LEP *variants investigated with calving interval would question the potential importance of these variants, or indeed leptin itself, in selection for improved fertility in the Holstein-Friesian dairy cow. This information may be useful in marker assisted selection, or some derivative of such (e.g,. genomic selection placing greater prior emphasis on known QTLs), to increase the accuracy of selection, in particular milk composition, thereby increasing genetic gain.

## Methods

### DNA extraction, Genotyping and Study population

Genomic DNA from frozen semen straws was extracted using the following procedure: thawed semen was washed twice in phosphate buffered saline (pH 7.4). Cell pellets were harvested by centrifugation and re-suspended in 450 μL of pre-warmed extraction buffer (10 mM Tris pH 8, 10 mM EDTA pH8.0, 1% SDS, 100 mM NaCl). Fifteen μL of 2-Mercaptoethanol was added. Samples were incubated at 55°C for 15 minutes followed by the addition of 10 μL Proteinase K (20 mg/ml). Lysis occurred following an overnight incubation at 60°C. DNA was extracted using the Maxwell^® ^instrument (Promega Corp., Madison, WT, USA), according to the manufacturer's instructions. For SNP discovery, 1.656 kb of the *LEP *regulatory region was amplified by polymerase chain reaction (PCR) from genomic DNA of 14 Holstein-Friesian sires using four pairs of oligonucleotide primers designed based on the published sequence (GenBank AB070368). Primer pair -1656F 5' GTGGATGCTACTGCCTCTATT 3' and -929R 5' GCCTGGTTGTTTTGCTTTTA 3', using an annealing temperature of 56°C with 2 mM MgCl_2_, amplified a product of 720 bp. Primer pair -1358F 5' AAGTCCCCTGTAGATGTTTTTATG 3' and -579R 5' CGGGTCCGTTTTGTTCAC 3', using an annealing temperature of 54°C with 1.5 mM MgCl_2, _amplified a product of 779 bp. Primer pair -630F 5' GCAACGCACGGGGCTATCAATG 3' and -446R 5' GCCTGGCCTGGAAAATCACACCT 3', using an annealing temperature of 68°C with 1.5 mM MgCl_2, _amplified a product of 206 bp. Primer pair -473F 5' CTTACCCCTCCACACCATCATCAA 3' and + 33R 5' GAGCCGGGCACTTACCT 3', using an annealing temperature of 60°C with 1.5 mM MgCl_2_, amplified a product of 522 bp. PCR amplicons were sequenced bi-directionally by Cogenics (North Carolina, U.S.A) and the data analysed using the Lazergene 6 suite of programs (DNAStar Inc., Madison WI, USA). Where sequencing reads were consistently poor, PCR amplicons were cloned in order to sequence each allele separately. For cloning, the TOPO Cloning kit (Invitrogen Ltd., Paisley, UK) was used according to the manufacturers' instructions and for each cloned amplicon, plasmid DNA from at least 4 clones was sequenced.

SNP genotyping analysis was performed by Sequenom^® ^using the iPLEX Gold assay on a MassARRAY^® ^Platform (http://www.sequenom.com). In total, 10 SNPs (LEPR-T945M, LEP-2470, LEP-1609, LEP-1457, LEP-1238, LEP-963, LEP-580, Y7F, R25C and A80V) were selected for genotyping in 848 Holstein-Friesian sires with progeny in Ireland. These sires originated either directly or indirectly (e.g., sons of sires) from international breeding programs and were representative of sires used in Ireland in recent years. Polymorphisms LEP-2470 (also referred to as UASMS-2) [13], LEP-1457 [20], LEP-1238 (also referred to as UASMS3) [13], LEP-963 [20] and LEP-580 [20] are designated according to their distance upstream from the transcription start site. SNP T7F is located in exon 2 and results in a tyrosine to phenylalanine substitution at amino acid position 7 within the peptide signal sequence [16]. The C to T SNP of R25C results in an arginine to cysteine substitution at amino acid 25 in the α-helix of the leptin protein [50]. A80V SNP in exon 3 substitutes an alanine for valine at amino acid position 80 [53]. The LEPR-T945M is a T to C polymorphism in exon 20 of LEPR which results in a threonine for methionine substitution at residue 945 [30]. Genomic DNA from twenty-five animals was genotyped in duplicate for each SNP. Concordance across the 10 SNPs and all duplicates was 100%. TESS (http://www.cbil.upenn.edu/tess) was used to search for putative transcription factor binding sites.

### Genotypic and phenotypic data

A measure of linkage disequilibrium r^2 ^[54] was calculated between each pair-wise leptin combination of the segregating SNPs. PHASE V2.1 [55,56] was used to reconstruct haplotype probabilities. Daughter yield deviations (DYD) and PTA, as well as associated reliabilities for a range of performance traits evaluated by the Irish Cattle Breeding Federation in January 2009, were available for inclusion in the analysis. The range of phenotypic traits could be broadly categorised into milk energy output traits (milk production), reproduction traits (calving interval, calving difficulty, perinatal mortality and gestation length), and energy storage traits (angularity, BCS, carcass fat). Models used in genetic evaluations in Ireland, as well as variance components, are outlined by Berry *et al *[57]. DYDs for 305-day milk, fat and protein yield as well as geometric mean SCS (log_e _somatic cell count) are estimated in Ireland using a repeatability animal model across the first five lactations. DYDs expressed on the scale of PTA were used in this study. A DYD is the weighted average of the deviation of a bull's daughters from their contemporaries adjusted for merit of their dams. A PTA is the average genetic merit an animal is expected to transmit to its offspring. PTA for calving interval and survival are estimated using a multi-trait animal model, including data from the first three lactations. PTA for milk yield is used to adjust survival for differences in genetic merit of milk yield; hence, this survival trait is functional survival. PTAs for progeny carcass fat score, measured at slaughter, are estimated in a multi-trait animal model that includes carcass weight, carcass conformation, cow carcass weight, weaning weight, live-weight of the animal between 300 and 600 days of age, feed intake, and skeletal and muscular linear traits. Male progeny slaughtered between 300 and 1,200 days of age and female progeny slaughtered between 300 and 875 days of age are included in the evaluation of carcass fat. Genetic evaluations for angularity and BCS, both subjective measures of subcutaneous fat depots, are undertaken as part of a joint evaluation in the UK and Ireland. The PTAs are standardised to the mean and standard deviation of the base population. Perinatal mortality is a dichotomous variable scored by farmers as calf dead at birth or within 24 hours [58]; calving difficulty, also scored by farmers is scored on a scale of 1 (easy) to 4 (veterinary assistance required). Gestation length is the number of days from service to birth of the calf. Genetic merit for perinatal mortality and gestation length are estimated using univariate animal linear mixed models. Genetic merits for direct and maternal calving difficulty are estimated using an animal-dam linear mixed model.

PTAs were de-regressed using the procedure outlined by Berry *et al *[59] to remove the contribution of pedigree to the estimated genetic merit of the animal. Parental contribution to the reliability of each DYD or PTA was removed using the approach of Harris and Johnson [60] and only sires with a reliability, less parental contribution, of > 60% were retained for inclusion in the association analysis. A total of 742 sires fulfilled these criteria for inclusion in the analysis of milk, fat and protein yield as well as milk fat and protein concentration; the number of sires included in the association analysis with SCS, calving interval and survival was 703, 501, and 477 respectively. The number of sires included in the analysis of angularity, BCS and carcass fat was 521, 504 and 446, respectively. The number of sires for direct calving difficulty, maternal calving difficulty, perinatal mortality and gestation length was 575, 506, 201 and 614 respectively.

### Analysis

The association between each SNP and performance was quantified using weighted mixed models in ASREML [61] with individual included as a random effect, and average expected relationships among individuals accounted for through the numerator relationship matrix. Year of birth (divided into 5 yearly intervals) and percent Holstein of the individual sire were included as fixed effects in the model. In all instances the dependent variable was the DYD or deregressed PTA of interest, weighted by its respective reliability less the parental contribution. Genotype was included in the analysis as a continuous or a class variable coded as the number of copies of a given allele. In a separate analysis, the probability of each haplotype for the individual was included as fixed effect in the weighted animal mixed model with year of birth of the bull and Holstein proportion also included as fixed effects; the haplotype with the greatest frequency was not included in the model to avoid linear dependencies among haplotype effects. Similarly when genotype was included in the model as a class effect one of the genotypes was set to zero.

## Authors' contributions

LG designed and coordinated the study and participated in manuscript drafting. SB participated in the design of the study and drafting the manuscript. BMK performed sequence analysis. SMW coordinated and prepared DNA samples for genotyping. MJC reviewed sequencing data and helped draft the manuscript. DB provided statistical analysis and participated in manuscript drafting. All authors have read and approved the final manuscript.

## Supplementary Material

Additional file 1 **SNPs with phenotypic associations in Tables **3**, **4**and **5**are included in the model as a class effect**. ^a^Referent class is the homozygous genotype not presented for the respective SNP. Significance of genotype association with the performance variable: ^† ^= P < 0.10; * = P < 0.05. Standard error in parenthesis. BCS: Body Condition Score. SD Units: Standard deviation Units Click here for file
