# Supplementary material for: Association of bovine leptin polymorphisms with energy output and energy storage traits in progeny tested Holstein-Friesian dairy cattle sires
Source: BMC Genet. 2010 Jul 29;11:73. doi: 10.1186/1471-2156-11-73 (PMC2920856; doi:10.1186/1471-2156-11-73)
Supplement: Additional file 1 — SNPs with phenotypic associations in Tables 3, 4and 5are included in the model as a class effect. aReferent class is the homozygous genotype not presented for the respective SNP. Significance of genotype association with the performance variable: † = P < 0.10; * = P < 0.05. Standard error in parenthesis. BCS: Body Condition Score. SD Units: Standard deviation Units [file 1471-2156-11-73-S1.DOC]

**Additional File 1 SNPs with phenotypic associations in Tables 3, 4 and 5 are included in the model as a class effect**

| **SNP** | **Genotype^a^** | **Milk yield (kg)** | **Protein percent (%)** | **Calf perinatal mortality (%)** |
| --- | --- | --- | --- | --- |
| LEP-2470 | C/T | 27.84 (14.9) | -0.011 (0.0055) ^†^ | 0.28 (0.1465) ^†^ |
|  | T/T | 2.87 (52.66) | -0.023 (0.0203) ^†^ | 0.711 (0.4947) ^†^ |

| **SNP** | **Genotype^a^** | **Fat percent (%)** | **Protein percent (%)** | **SCS (units)** | **Direct caving difficulty (%)** | **Gestation length (days)** | **Calf perinatal mortality (%)** |
| --- | --- | --- | --- | --- | --- | --- | --- |
| LEP-1238 | C/G | -0.002 (0.0142) ^†^ | -0.006 (0.0069) ^†^ | 0.015 (0.0128) | 0.333 (0.2347) | 0.271 (0.1452) ^†^ | 0.212 (0.1957) |
|  | G/G | -0.026 (0.0155) ^†^ | -0.015 (0.0075) ^†^ | 0.023 (0.0137) | 0.516 (0.2587) | 0.352 (0.1626) ^†^ | 0.34 (0.1998) |

| **SNP** | **Genotype^a^** | **Milk yield (kg)** | **Fat percent (%)** | **Protein percent (%)** | **Direct caving difficulty (%)** | **Gestation length (days)** |
| --- | --- | --- | --- | --- | --- | --- |
| LEP-963 | C/T | -9.91 (13.46) | 0.020 (0.0102) ^†^ | 0.006 (0.005) | -0.275 (0.1738) * | -0.271 (0.108) ^†^ |
|  | T/T | -36.87 (20.26) | 0.028 (0.0153) ^†^ | 0.015 (0.0074) | -0.618 (0.2539) * | -0.636 (0.1603) ^†^ |

| **SNP** | **Genotype^a^** | **Milk yield (kg)** | **Protein yield (kg)** | **Angularity (SD Units)** | **BCS (SD Units)** | **Gestation length (days)** |
| --- | --- | --- | --- | --- | --- | --- |
| Y7F | A/T | -35.93 (24.97) | -1.2 (0.69) | -0.69 (0.27)* | 0.32 (0.22) | -0.385 (0.2021) |
|  | T/T | -103.8 (93.43) | -2.83 (2.58) | -1.74 (0.85)* | 0.56 (0.83) | -0.361 (0.647) |

| **SNP** | **Genotype^a^** | **Fat percent (%)** | **Protein percent (%)** | **Direct caving difficulty (%)** | **Gestation length (days)** |
| --- | --- | --- | --- | --- | --- |
| R25C | C/T | 0.022 (0.010) ^†^ | 0.007 (0.0049) | -0.244(0.1738) ^†^ | -0.155 (0.1078)* |
|  | T/T | 0.025 (0.0152) ^†^ | 0.015 (0.0075) | -0.571 (0.2564) ^†^ | -0.387 (0.1619)* |

| **SNP** | **Genotype^a^** | **Survival (%)** |
| --- | --- | --- |
| A80V | C/T | -0.285 (0.1822) |
|  | T/T | -0.648 (0.361) |

^a^Referent class is the homozygous genotype not presented for the respective SNP. Significance of genotype association with the performance variable: ^†^ = P<0.10; * = P<0.05

Standard error in parenthesis

BCS: Body condition score

SD Units: Standard Deviation Units
